# Quality newborn care in East New Britain, Papua New Guinea: measuring early newborn care practices and identifying opportunities for improvement

**DOI:** 10.1186/s12884-022-04735-7

**Published:** 2022-06-01

**Authors:** Alyce N. Wilson, Pele Melepia, Rose Suruka, Priscah Hezeri, Dukduk Kabiu, Delly Babona, Pinip Wapi, Naomi Spotswood, Meghan A. Bohren, Joshua P. Vogel, Angela Kelly-Hanku, Alison Morgan, James G. Beeson, Christopher Morgan, Lisa M. Vallely, Edward J. Waramin, Michelle J. L. Scoullar, Caroline S. E. Homer

**Affiliations:** 1 Maternal, Child and Adolescent Health Program, Burnet Institute, Melbourne, Australia; 2 Nossal Institute for Global Health, School of Population and Global Health, University of Melbourne, Melbourne, Australia; 3 Healthy Mothers, Healthy Babies, Burnet Institute, Kokopo, Papua New Guinea; 4 St Mary’s Hospital, Kokopo, Papua New Guinea; 5 Nonga General Hospital, Rabaul, Papua New Guinea; 6 Department of Medicine, University of Melbourne, Melbourne, Australia; 7 Department of Paediatrics, Royal Hobart Hospital, Hobart, Tasmania Australia; 8 Gender and Women’s Health Unit, Centre for Health Equity, School of Population and Global Health, University of Melbourne, Melbourne, Australia; 9 Papua New Guinea Institute for Medical Research, Goroka, Papua New Guinea; 10 Kirby Institute, University of New South Wales, Kensington, NSW Australia; 11 Global Financing Facility, World Bank Group, Washington, DC USA; 12 Jhpiego, the Johns Hopkins University affiliate, Baltimore, USA; 13 Population and Family Health, National Department of Health, Port Moresby, Papua New Guinea; 14 Department of Paediatrics, University of Melbourne, Melbourne, Australia; 15 School of Population and Global Health, University of Melbourne, Melbourne, Australia

**Keywords:** Quality care, Maternal and newborn health, Newborn care, Papua New Guinea

## Abstract

**Background:**

Renewed attention and investment is needed to improve the quality of care during the early newborn period to address preventable newborn deaths and stillbirths in Papua New Guinea (PNG). We aimed to assess early newborn care practices and identify opportunities for improvement in one province (East New Britain) in PNG.

**Methods:**

A mixed-methods study was undertaken in five rural health facilities in the province using a combination of facility audits, labour observations and qualitative interviews with women and maternity providers. Data collection took place between September 2019 and February 2020. Quantitative data were analysed descriptively, whilst qualitative data were analysed using content analysis. Data were triangulated by data source.

**Results:**

Five facility audits, 30 labour observations (in four of the facilities), and interviews with 13 women and eight health providers were conducted to examine early newborn care practices. We found a perinatal mortality rate of 32.2 perinatal deaths per 1000 total births and several barriers to quality newborn care, including an insufficient workforce, critical infrastructure and utility constraints, and limited availability of essential newborn medicines and equipment. Most newborns received at least one essential newborn care practice in the first hour of life, such as immediate and thorough drying (97%).

**Conclusions:**

We observed high rates of essential newborn care practices including immediate skin-to-skin and delayed cord clamping. We also identified multiple barriers to improving the quality of newborn care in East New Britain, PNG. These findings can inform the development of effective interventions to improve the quality of newborn care. Further, this study demonstrates that multi-faceted programs that include increased investment in the health workforce, education and training, and availability of essential equipment, medicines, and supplies are required to improve newborn outcomes.

**Supplementary Information:**

The online version contains supplementary material available at 10.1186/s12884-022-04735-7.

## Background

Over the last few decades, there have been major improvements in child survival, yet gains for newborns are comparatively lagging [1]. Neonatal deaths in the first 28 days of life now account for 47% of all under 5 deaths globally [2]. The majority of these deaths occur in low- or middle-income countries (LMICs) [3], and are predominantly due to three preventable causes: intrapartum-related deaths including asphyxia, complications due to prematurity and neonatal infections [4]. Newborn survival has been described as a ‘sensitive marker of a health system’ [5], meaning whether a health system can respond to the health needs of its smallest and youngest citizens.

More than 40% of all newborn deaths occur in the first 24 hours of life, highlighting the critical role improving the quality of care around the time of labour and birth can have to greatly improve newborn survival [5]. Quality maternal and newborn care includes the provision of evidence-based care by skilled providers during pregnancy and birth in a respectful and supportive environment [6]. The World Health Organization (WHO) recommends that all newborns receive four simple, low-cost interventions [7–9]: immediate and thorough drying [10], immediate skin-to-skin contact [11, 12] delayed cord clamping [13] and initiation of breastfeeding in the first hour [14]. These interventions have been shown to make a significant difference to newborn survival, especially for low birth weight infants [15]. Investing in, and improving, newborn survival also provides economic benefits and is cost-effective [16]. For example, global analyses estimate that the provision of quality care for all women and newborn babies in health facilities could prevent approximately 531,000 stillbirths and 1.325 million neonatal deaths worldwide at a cost of US$4.5 billion/year (US$0.9 per person) [16]. However, in order to achieve these gains, the availability of essential medicines and supplies and a competent and skilled health workforce working within an enabling environment [17] is critical [5]. An enabling environment refers to an environment that has the appropriate infrastructure, profession and system-level integration needed for maternity care providers, particularly midwives, to effectively provide their full scope of practice [17].

Papua New Guinea (PNG) is the most populated country in the Pacific with a population of more than eight million. It is a lower-middle-income country with high rates of maternal and newborn morbidity and mortality. The most recent PNG Demographic Health Survey estimated the neonatal mortality to be approximately 20 per 1000 live births [18], almost double the Sustainable Development Goal (SDG) target of 12 deaths per 1000 live births by 2030 [19]. Stillbirth rates are estimated to be between 20 to 30 per 1000 live births [20]. A review of 35 early neonatal deaths among 2499 live births across two provinces in PNG (East New Britain and Madang) identified several preventable causes and avoidable factors associated with these deaths, including insufficient resources at health facilities, poor quality of care during labour and birth, and poor management of high-risk newborns, including neonatal resuscitation [21]. Similarly, a review of stillbirths across the same two provinces identified at least one avoidable factor for 95% of the 59 stillbirths reviewed, included health personnel-associated factors related to poor intrapartum care and lack of early diagnosis and intervention of complications [22].

In PNG, a chronic shortage of health providers, especially midwives, lack of essential medicines and supplies, under-equipped facilities, an underfunded health system, socio-cultural factors and difficult geography all contribute to high neonatal mortality and stillbirth rates [23]. A recent health service review estimated that there are only nine health providers per 10,000 people in PNG [24], well below the World Health Organization (WHO) recommendation of 44 per 10,000 people for universal health coverage [25]. In addition, only 41% of all health facilities had running water to the birth room and less than half (48%) of all pregnant women had one antenatal care visit, with only 36% of women having a facility birth [24]. It is likely that the COVID-19 pandemic has exacerbated care access and health workforce issues even further. These factors represent substantial hurdles to overcome in improving the quality of maternal and newborn care in PNG, especially during the intrapartum and early newborn period.

PNG has been previously identified by the World Health Organization (WHO) as a priority country for improving newborn survival, as it is one of eight high burden countries that account for over 96% of neonatal deaths in the Western Pacific Region [26]. In an effort to reduce neonatal deaths, WHO and UNICEF developed an Action Plan for Healthy Newborn Infants in the Western Pacific Region (2014–2020) [27]. This plan identified an urgent need for priority countries to incorporate and scale up early essential newborn care into routine care practices [27]. Early essential newborn care has been associated with reduced neonatal sepsis, increased rates of skin-to-skin post birth, higher rates of exclusive breastfeeding at discharge, and lower neonatal intensive care unit admissions for both vaginal and caesarean section born babies [28, 29].

In a 2017 progress review of action plan implementation, only 29% of health services that provide childbirth services in PNG had introduced early essential newborn care practices, well below the 80% benchmark [26]. However, initial pre- and post- testing results from PNG facilities that had received early essential newborn care coaching demonstrated promising findings with improvements in hand hygiene and breathing/non-breathing baby management [30]. A significant amount of work has been undertaken in PNG and the Western Pacific region more broadly to improve early newborn care [15, 26, 27, 30]. For PNG to meet international [5, 19] targets for preventable newborn deaths and stillbirths, there is a need for continued attention and investment in efforts to improve the quality of care during labour, birth and the early newborn period (first day and week). These efforts need to be driven and informed by high-quality local data, in addition to the global evidence base. Understanding how to meaningfully measure early essential newborn care practices (whether practices are performed or not) in PNG is essential to inform quality improvement actions, in addition it is equally important to examine the quality of the care (whether care is provided in a timely, efficient, effective, safe, person-centered and respectful manner).

## Methods

### Aim, study design and setting

The aim of this study was to measure the provision of early newborn care and identify opportunities for improvement in a rural province of PNG.

This paper reports on a nested newborn care analysis within a larger mixed-methods quality improvement study - known as the ‘Gutpela Helt Sevis Stadi’ (in English: ‘Quality Health Services Study’) – the overall aim of which was to improve the quality of maternal and newborn care in five facilities in East New Britain, PNG. The Gutpela Helt Sevis Stadi was co-designed and implemented in partnership with local health services and community members in accordance with a Partnership Defined Quality (PDQ) approach [31]. Ethical approvals were received from relevant authorities in PNG and Australia (MRAC 19.16 and Project No. 267/19).

East New Britain is a rural province located in the New Guinea Islands region of PNG. Around 400,000 people live in East New Britain [32] and approximately 10,000 babies are born in the province each year [33], 60% of these births occur in health facilities [32]. Like the rest of PNG, East New Britain has a significant shortage of health providers, especially with obstetric and midwifery skills, with only 60 health providers per 10,000 people [33]. Rates of low-birth weight babies (< 2500 g) in East New Britain are slightly higher than the national average (7.3% vs 6.9%) [34]. Other local determinants of poorer newborn health outcomes include high rates of unintended pregnancy, low levels of family planning [35] and a high burden of reproductive tract infections [36]. In 2018, the year prior to commencement of this study, an early essential newborn care workshop was held in East New Britain with over 20 participants attending from various health facilities in East New Britain Province. As a result of the workshop, newborn care practices including breastfeeding within one hour of birth, skin-to-skin contact and newborn resuscitation practices were added to hospital record keeping systems.

### Participating facilities, women and providers

Five health facilities were nominated by the Provincial Health Authority (who manage health services in the province) to participate in the study, including a tertiary referral hospital, one secondary hospital, one rural hospital, one health centre and one community health post. The PHA nominated these facilities to participate in the study because they collectively provide approximately 70% of the facility-based birthing services in the province, have been involved in previous research [13, 14, 32], represent a combination of Government- and faith-based organisation-run services, and capture the referral pathway from a remote community health post to a tertiary referral hospital. Facilities included Level I (provide care to well newborns) and II (provide care to babies born at or after 32 weeks and weigh more than 1500 g) neonatal care facilities [37].

Women were eligible for labour observation if they were over 16 years of age and admitted for childbirth, and not transferred to another hospital or taken straight to the operating theatre (as the purpose was to examined care provided during labour and birth). Eligible women were provided with information about the observation and recruited if they provided verbal and written informed consent.

For the qualitative interviews, women and maternity care providers were purposively recruited. Women were recruited from facility postnatal wards, the day after birth. Researchers approached women, provided information about the study face-to-face with potential participants and asked them if they were interested in participating. Women were eligible if they were over 16 years of age and had recently given birth in one of these five facilities. Maternity care providers including midwives, nurses, doctors (obstetrician and gynaecology doctors), community health workers (CHWs) and Health Extension Officers (HEOs), were also informed about the study via face-to-face discussions and invited to participate. In PNG, CHWs complete two years of training and are specifically trained to work in rural areas. HEOs undertake a four-year training program and have skills in clinical and administrative practice. Both CHWs and HEOS, can provide antenatal, intrapartum and postnatal care. For the purposes of this study, maternity care providers referred to any health workers (midwives, doctors, nurses, CHWs, HEOs) providing labour and birth care to women during the labour observation period. All participants that were approached agreed to take part and no participants dropped out. Women and maternity providers who were observed during labour observations were not the same as those interviewed.

### Study coordination and data collection

Data collection methods included facility audits, labour observations and interviews with women and maternity care providers. Data collection took place between September 2019 and February 2020. A facility audit tool (Supplementary 1) was developed to assess the quality of maternal and newborn care drawing on existing validated tools [38, 39], clinical guides [15] and frameworks [6, 19]. The audit tool was piloted in health facilities in East New Britain, discussed with health workers and refined following multiple rounds of testing and dialogue. Audits reviewed items relevant to newborn care including hospital layout and utilities, workforce and rostering, availability of protocols and guidelines, auditing practices and medicines and equipment availability. Facility audits were conducted by trained research officers in each of the five health facilities, with information clarified with Officers in Charge (senior management staff) where needed.

Labour observations were conducted in four of the five facilities using a bespoke observation tool adapted from validated tools [40–43] to observe labour, birth and immediate postnatal care (Supplementary 2). The fifth site, a community health post, was excluded from labour observations as no births had taken place at that site in the 18 months prior to data collection. We still included the community health post in the facility audit as the PNG National Department of Health recognises community health posts as facilities capable of providing childbirth services [44]. Observations were conducted between 7 am and 6 pm by female researchers who continuously observed women during labour and birth, until one hour postpartum. These female researchers had relevant clinical backgrounds and received two weeks of targeted training before data collection. Researchers were instructed to only observe care practices and not to assist with the care of the women, however, they were advised to call for midwifery or medical assistance if needed. Research officers collected facility audit and labour observation data using REDCap [45] software installed on electronic tablets.

Individual face-to-face interviews were conducted with women and care providers to gain an in-depth understanding of newborn care practices. Interviews were conducted by experienced researchers (PM, RS, PH, DK) in the participant’s preferred languages using semi-structured interview guides (Supplementary 3) – generally in English for maternity providers, and Tok Pisin (national language ‘Pidgin’ spoken throughout PNG) or Tok Ples (local languages) for women. Interview guides were piloted with community members and health providers prior to data collection. The pilot process involved multiple rounds of testing and dialogue with both interviewers and interviewees to ensure appropriateness of questions and responses elicited. Interviews generally lasted between 30 to 60 minutes and were conducted in quiet, private locations away from the maternity wards, such as in a private room in the health facility or under a tree outside. Interviews were transcribed verbatim into Tok Pisin and then English to ensure accurate translation of meaning.

### Data analysis

Facility audit and labour observation data were extracted from REDCap software into Microsoft Excel spreadsheets for descriptive analysis using StataSE 15 software [46]. Univariate analyses were conducted using variables from facility audit and labour observation data. Variables included facility characteristics, utilities, medicines, equipment, supplies and newborn care practices. For example, facility characteristics included variables, such as, labour ward, special care nursery, postnatal ward, whilst utilities included hygiene and sanitation and power supply among other variables. Labour observation data included babies being immediately dried and placed on their mother’s chest after birth, delayed cord clamping, and early breastfeeding initiation. Audit data was cross-checked and validated with local clinicians (PW and DB). Results are presented as percentages and grouped into three categories: i) Green – high coverage (≥80%), ii) Orange – moderate coverage (60–80%), and iii) Red – poor coverage (≤60%) (Tables 2 and 3).

To ensure that results presented focused on key newborn care indicators, data analysis was informed by the *Every Newborn Action Plan* [5], *WHO Standards for Improving Quality of Maternal and Newborn Care in Health Facilities* [6], and the *Early Essential Newborn Care: Clinical Practice Pocket Guide* [15]. Interview data were analysed using qualitative content analysis [47] to determine the presence of key words, phrases and concepts related to the provision of newborn care and essential newborn care practices. NVivo software was used to manage qualitative data [48]. Segments of the transcripts related to newborn care were identified and extracted. Text within these segments were coded by a process of selective reduction – this enabled the researchers to focus on and code specific words, phrases and concepts related to newborn care provision [49].

Coding of transcripts was conducted by researchers in PNG and Australia (AW, RS, PH, RM, DK); three of these researchers have clinical backgrounds in medicine, nursing and community health. Researchers compared and cross-checked codes to ensure validity, consistency and coherency of codes [49]. Throughout the coding process, the team used a flexible approach to allow for the identification and analysis of concepts related to essential newborn care practices. We triangulated the data by data source, primarily from the labour observations and qualitative data, to explore how newborn care practices were actually implemented with respect to the key newborn care practices specified in the *Every Newborn Action Plan* [5]. These practices included immediate and thorough drying, delayed cord clamping, immediate skin-to-skin and early initiation of breastfeeding. Triangulation enabled us to bring together multiple data sources, providing a richer assessment of newborn care [50, 51]. In addition, we were able to compare and contrast the quantitative results with the qualitative data to allow verification, validation and greater exploration of study findings [52].

## Results

We conducted five facility audits, 30 labour observations (in four of the five facilities) and interviewed 13 women and nine health providers about early newborn practices. All women and providers approached for an interview agreed to participate. Health providers interviewed included five midwives, three community health workers and one nurse. Of the women interviewed, seven had given birth before and two were first-time mothers. Women were aged between 21 and 30 years, education levels ranged from primary school through to college and all were partnered. Almost all women had vaginal births; one had a caesarean section.

Across four of the five facilities (excluding the remote community health post), 5988 live births occurred in the year the audit was conducted (2019) (Table 1). No babies were born in the community health post. There were 89 early neonatal deaths (neonatal deaths within the first seven days of life) and 110 stillbirths, resulting in a perinatal mortality rate of 32.2 per 1000 total births (Table 2). Of these stillbirths, 44 were reported to be intrapartum and 66 antenatal. Two of the five (40%) facilities (tertiary and secondary hospitals) regularly held morbidity and mortality audit meetings.

**Table 1:** Description of facilities, birth rates and staffing across the five study sites

Facilities	Average number of births per month (pm) and per annum (pa)	Maternity workforce	Audit conducted	Qualitative interviews conducted	Labour observations conducted
**Community 1 – Tertiary Hospital**	180 pm 2160 pa	31 (4 doctors, 17 midwives, 6 nurses, 4 CHWs)	Yes	7	10
**Community 2 – Secondary Hospital**	200 pm 2400 pa	19 (2 doctors, 7 midwives, 2 nurses, 8 CHWs)	Yes	6	10
**Community 3 – Rural Hospital**	80 pm 960 pa	5 (2 midwives, 2 HEOs, 1 CHW)	Yes	4	5
**Community 4 – Health Centre**	39 pm 468 pa	24 (2 midwives, 1 HEO, 9 nurses, 12 CHWs)	Yes	4	5
**Community 5 – Community health post**	0	2 (1 nurse and 1 CHW)	Yes	–	0
**Total**	499pm 5988 pa	81	5	21	30

**Table 2:** Perinatal indicators and audits

Perinatal indicators	Across 4 facilities* ***N*** = 4
Average number of live births/year	5988
Number of facility-based early neonatal deaths in past 12 months	89
Number of stillbirths in past 12 months	110
Number of intrapartum stillbirths	44
Number of antenatal stillbirths	66
Facility perinatal mortality rate (per 1000 total births)	32.2
Number of facilities that meet to discuss neonatal and stillbirth morbidity and mortality cases (conduct clinical audits)	2

*Data source: Facility audits*

*Remote health post excluded

### Facility infrastructure

Across the five facilities, there were 81 health providers working in maternity care, including 28 midwives, 26 community health workers, 18 nurses, six doctors and three health extension officers (Table 1). The majority of staff were based in the tertiary hospital, which also had the largest number of midwives. Rosters with clearly displayed names of staff members and shifts were visible in four of the five facilities. The median time to the Provincial hospital by vehicle was one hour, ranging from 45 minutes to two hours. All facilities had access to a functioning ambulance, a labour ward and a postnatal ward. All facilities were open 24 hours a day, except the remote health post which had variable opening hours, generally 4 hours per day. Two facilities (both Level II neonatal care facilities) [37] (40%) had an operating theatre, pharmacy, laboratory, neonatal resuscitation beds and special care nursery. Across the five facilities, women’s average length of stay in hospital after a vaginal birth was 24 hours. All facilities had paper-based record systems.

### Local policies

One (20%) facility had a referral pathway protocol available and visible, and one had an established protocol for mothers and babies to ‘room in’ together. No facilities had infant formula company materials visible nor did mothers receive products or gifts from formula companies. Formula use was not examined.

### Utilities

At the time of the audit (pre-COVID-19 period), four (80%) facilities had running water and three (60%) had soap available. Three (60%) had alcohol hand rub available. All facilities had waste disposal systems in place. Four (80%) of the facilities received electricity via the national grid, and all had access to generators if needed. Electricity was functioning in four (80%) of the facilities at the time of the audit. Solar power was not present in any facilities. Regarding policies, protocols and guidelines, four (80%) of the facilities had PNG management guidelines in obstetrics and gynaecology available, whist three (60%) had paediatric guidelines available.

### Equipment and medicines

Availability of essential equipment and medicines for newborns varied widely across the facilities (Table 3). Four of the five (80%) facilities had common antibiotics (amoxicillin, ampicillin, ceftriaxone, penicillin G) and antimalarial agents. Three (60%) had supplies of eye antimicrobials, gentamicin, hepatitis vaccine and Vitamin K. Only one (20%) had supplies of the BCG vaccine and rapid malaria diagnostic tests. Three (60%) facilities had oxygen available, whilst four (80%) facilities had a newborn bag valve mask (self-inflating). All facilities had clean towels for drying and covering the baby, four (80%) had blankets and umbilical cord ties. All facilities had digital thermometers and four (80%) had baby-weighing scales. One (1) cubic centimeter (cc) and 3 cc syringes were available in all facilities. Feeding cups were only available in one facility.

**Table 3:**
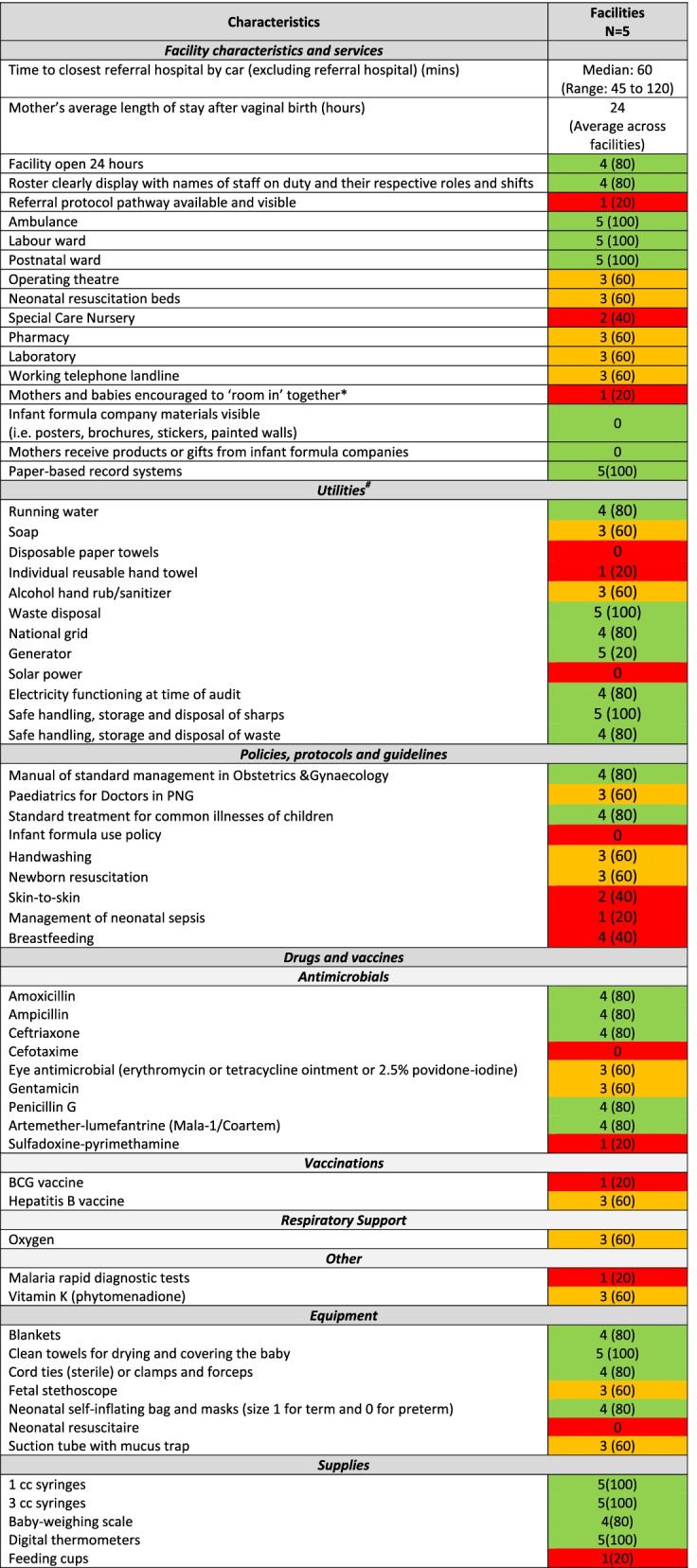
Facility characteristics and availability of essential equipment and supplies (Adapted from WHO EENC Clinic Practice Pocket Guide [15], WHO Quality Maternal and Newborn Care Standards [6], Columbia University Averting Maternal Death and Disability [38] and JHPIEGO Health Facility Assessment Tool [39])

*Data source: Facility audits*

*Not measured: Dextrose 10%, Plain Ringer’s lactate or normal saline, Oral Rehydration Salt, Sterile water for injection, Zinc,* Bonnets, mittens and socks*,* Feeding tubes (Fr 5 and 8)*,* Support binders for skin-to-skin

*Green = high coverage (> 80%), Orange = moderate coverage (60–80%), Red = poor coverage (< 60%)

*^The practice of rooming-in is defined by the World Health Organization as a “hospital practice where postnatal mothers and infants stay together in the same room for 24 hours a day from the time they arrive in their room after birth”*

*# Utilities located in the labour ward/newborn unit, available and fully functional at the time of the audit*

### Newborn care practices

We observed key newborn care practices as part of the 30 labour observations conducted in four facilities (80%) (Table 4). Newborn care practices were further discussed through interviews with women and health providers (Table 5). In all observations (97%) women had a skilled birth attendant present at the time of birth. In most observations (77%), birth attendants (midwives, doctors, nurses, CHWs) made changes to adjust the room temperature for the women’s comfort i.e. turning on/off fans/air conditioning. Newborn resuscitation equipment was prepared (i.e. birth pack, towels, self-inflating bag and mask), with hands washed and gloves donned, prior to birth for 43% of the births observed, and newborn bag and masks were checked during 37% of observations.

**Table 4:**
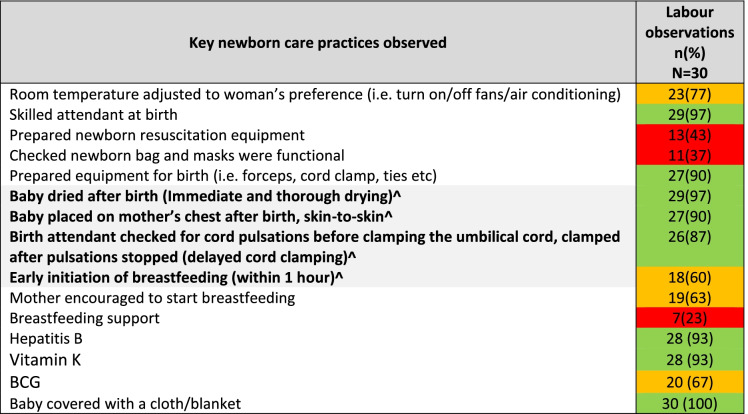
Key newborn care practices performed in health facilities in East New Britain, PNG (Adapted from WHO Quality Maternal and Newborn Care Standards [6] and Every Newborn Action Plan [5])

*Data source: Labour observations*

*Green = high coverage (> 80%), Orange = moderate coverage (60–80%), Red = poor coverage (< 60%)

*^Four elements of essential newborn care*

**Table 5:** Provision of essential newborn care practices described in interviews (Every Newborn Action Plan [5])

Essential newborn care practices	Sample of coded text
Immediate and thorough drying	*They came and wiped the baby and then lay it down on my chest. (Secondary hospital, Mother, Vaginal Birth, 2nd baby)* *They wipe the blood from the baby, and then they got a blanket and wrap it around the baby and put the baby in the cot. (Tertiary hospital, Mother, Vaginal Birth, 1st baby)* *After they put the baby on my tummy, they clean the baby [with a cloth], and also me the mother, they both clean both of us. (Tertiary hospital, Mother, Vaginal Birth, 3rd baby)* *We place the baby on the mother’s tummy and we have to wipe the baby’s face and mouth in case baby inhales or swallow particles. (Health centre, Community Health Worker)* *I wipe the face and skin and rub until the baby is dry and then put the baby on the mother’s chest. Okay, now I cover the baby with a blanket and the mother will hold the baby and they both will sleep so I will let the baby stay there for a while (Tertiary hospital, Midwife)*
Baby placed on mother’s chest after birth, skin-to-skin	*Baby lie on my chest for roughly thirty to forty minutes (Tertiary hospital, Mother, Vaginal Birth, 2nd baby)* *She got the baby and placed on my tummy (Rural hospital, Mother, Vaginal Birth, 6th baby)* *The baby cried when delivered and then was placed on my chest. (Secondary hospital, Mother, Vaginal Birth, 2nd baby)* *I didn’t have the opportunity to see my baby because I was in operation, maybe they operate on me, get the baby and they straight away gave it to the sister in charge of nursery … and they keep it in the nursery for one whole day until the next morning (Tertiary hospital, Mother, Married, Caesarean Section, 2nd baby)* *When the baby is born we remove the baby and put the baby on the mother’s chest (Community health post, Community Health Worker)* *When I deliver the baby, I firstly place baby on the mother’s belly and I tell her that her baby is a boy or a girl. I tell the mother to hug the baby and ask her if she is happy now that the pain is gone (Health centre, Community Health Worker)* *Having the baby on top of the mother’s chest, they’re skin to skin because it is very important that the immune, the warmth from the mother passes to the baby. We try our utmost best not to remove the baby from the mother … We can leave the baby lying there on the mother for more than 90 minutes. (Secondary hospital, Midwife)*
Delayed cord-clamping	*Previously when the baby is born we cut the umbilical cord straight away but currently it has changed … we wait for the pulsation of the cord to stop and we cut the cord. (Health centre, Community Health Worker)* *If we are very busy, we leave the baby on the mother’s chest and just wait for the cord to stop pulsating, clamp, cut … Just last month, I started realizing that lately we were not waiting for the cord to stop pulsating. As soon as the baby was born, the cord stopped pulsating immediately so I asked some trainee students, if they noticed what was happening. I mentioned this to them and they confirmed that ‘yes, it’s true, that they are no longer waiting for the cord to stop pulsating and they are cutting the cord straight away. (Tertiary hospital, Midwife)* *We wait if the cord is still pulsating; we don’t cut. We wait until the cord stops pulsating and then we cut, wrap the baby and leave the baby with the mother so that we can continue with the mother. (Rural hospital, Midwife)*
Early initiation of breastfeeding	*She [health provider] moves the baby close for breastfeeding and then she leaves us. (Rural hospital, Mother, Vaginal Birth, 4th baby)* *Later the baby is looking for breast milk. And I said, oh baby come out and looking for breast milk so I must feed the baby as soon as possible, and I give the baby breast milk. I know baby want to breastfeed and I breastfeed the baby quickly. I sleep the baby to my side on my hand and feed the baby with breast milk, and the baby started breastfeeding. (Secondary hospital, Mother, Vaginal Birth, 3rd baby)* *They [health providers] told me to breastfeed the baby, I must breastfeed the baby, when the baby keeps on sucking the breast milk will come. (Secondary hospital, Mother, Vaginal Birth, 1st baby)* *We tell the mother she can breast feed the baby if she sees that the baby is ready for feed. (Rural hospital, Midwife)* *We keep the baby with the mother for breast feeding, baby can breast feed and after that we give immunisation. (Rural hospital, Midwife)* *So after we clean the mother, we put the baby on her breast so that the baby can start to feed. While she is feeding the baby … We give advice on exclusive breast feeding, baby feeding, placenta, nutrition and diet, family planning and monthly immunization for the baby. Or if she feels any pain around her abdomen or pain during breastfeeding, then we reassure her and tell her, this is the involution of her uterus and also about her reproductive system. (Tertiary hospital, Midwife)*

With respect to the four essential elements of newborn care (skin-to-skin, delayed cord clamping, early breastfeeding initiation and thorough drying), in 90% of observations, babies were placed on their mother’s chest immediately after birth (skin-to-skin) and in 87% health providers practiced delayed cord clamping (at least 1 minute after birth). In almost all observations, babies (97%) were immediately and thoroughly dried after birth. In over half (60%) of the observations, babies commenced breastfeeding within one hour of birth. Whilst in 63% of observations, mothers were encouraged by health providers to start breastfeeding, in only 23% of the observations women received breastfeeding support (advice and guidance on good positioning and attachment). Of the 30 newborn observations (all singleton births), five needed support with breathing, all of these babies were rubbed/dried with a cloth. Of these five observations, delayed cord clamping was not observed for two babies, and immediate breastfeeding not observed for three. In most observations, babies received Vitamin K and Hepatitis B vaccination (93% for both), and 67% received the BCG vaccine.

We interviewed 21 women and health providers to validate essential newborn care practices recorded during labour observations (Table 5). With respect to immediate and thorough drying, women and health workers described how the baby was wiped clean, placed on the women’s chest and covered with a blanket. Some women noted that they themselves were also cleaned by their birth attendant. Skin-to-skin practices were consistently noted by women and health providers, who both described how once the baby was born they were immediately placed on the women’s chest. Some health providers also went on to describe why they did this and what the health benefits were for mother and baby. One mother, who had a caesarean birth, described how her baby was taken directly to the nursery after birth and she was not able to hold or be with the baby until the next morning. Health providers described delayed cord clamping practices and how they would wait for the cord to stop pulsating before they cut it. However, a midwife from the tertiary hospital noted that they had observed that this practice had dropped off lately. Mothers described how they started breastfeeding soon after birth, following cues from their baby and advice from their birth attendant. Health providers described how they would put the babies on a mother’s breasts so that the baby could start breastfeeding, followed by information and reassurance.

## Discussion

This study used a mixed-methods approach to measure early newborn care practices in five rural health facilities in East New Britain, PNG. We found a perinatal mortality rate of 32.2 perinatal deaths per 1000 total births and several barriers to quality newborn care, including an insufficient maternity workforce [53], infrastructure and utility limitations, and limited availability of many essential newborn medicines and equipment. The perinatal mortality rate for these four facilities was consistent with other perinatal mortality rates reported for PNG [20, 54]. Given that 40% of births in East New Britain occur outside of the facility [33], it is likely that the actual perinatal mortality rate in the province is much higher. Strengths identified from newborn observations included the presence of skilled birth attendants at most births and consistent provision of Hepatitis B vaccine and Vitamin K. We found, both by observation and interview, that many newborns received individual essential newborn care practices, such as, immediate skin-to-skin and delayed cord clamping.

Our findings are similar to another PNG study conducted in a different province in 2014, which examined the capacity of five facilities to provide quality newborn care and similarly found under-resourced facilities, electricity and power issues, insufficient basic equipment for the care of newborns and little or no supervisory support or professional development opportunities for staff [55]. A study which assessed the quality of newborn care in five facilities in a neighbouring Pacific island country, the Solomon Islands, found that whilst essential medicines were generally available [56], other barriers to quality newborn quality care were consistent with those identified in our study including workforce limitations, equipment supply, and lack of infection control measures. A subsequent study by the same research team involved implementation of an intervention to improve the quality of newborn care, specifically through training, equipment provision and health system organisation changes, finding enhanced skills and an improved workplace culture as a result of the training [57].

A sufficient, trained and motivated maternity care workforce is essential to improving the quality of newborn care. It is likely that the deficiencies in newborn care practices we found relate in part to the fact that the number of maternity providers in the five facilities are insufficient to meet the maternal and newborn health needs of the community, especially when staff shortages occur due to staff sickness, annual leave, education and training requirements. Caring for multiple women and babies, along with equipment and medicine shortages are additional constraints to providing essential newborn care. Although our data collection pre-dated the COVID-19 pandemic, the situation is likely to be worse when staff have been furloughed due to COVID-19 outbreaks. The health workforce required per population to meet a community’s sexual, reproductive, maternal, newborn, child and adolescent health needs is difficult to estimate [58]. There have been calls to move beyond universal benchmarks and consider the local population profile, demographics, fertility rates and epidemiology when determining the number of health providers needed [58]. There is also a need to consider equitable distribution of the workforce across urban, rural and remote areas and consider new ways to support and retain staff.

There is an urgent need to increase the number of midwives and other skilled birth attendants globally and in PNG [53, 59, 60]. Midwives are an especially important cadre for improving newborn care and when trained according to global standards and working within a supportive team and environment, midwives can meet at least 90% of a community’s sexual, reproductive, maternal and newborn health needs [58, 59]. However, it is not enough to increase numbers alone. Midwives need access to equipment, resources in an enabling environment, support from an interdisciplinary team and access to referral hospitals to provide quality care [59]. All health providers need access to ongoing education and training to maintain knowledge, skills and up-to-date practice, but moreover they need supervision and mentoring to grow and develop as health leaders who can lead and strengthen health services, and guide the next generation of health providers. In many LMICs, such as PNG, the shortage of health providers is so severe that there is often little capacity for qualified educators to provide this type of supervision and mentoring which is particularly critical for newly graduated cadres of health providers [61].

Strong and complete health information systems are necessary to provide valid and representative data for action and accountability in improving newborn care. Data can be used to provide a baseline assessment of newborn care, identify priority areas and track progress and effectiveness of quality improvement efforts. Data are also important for accountability and review processes [5]. No electronic record systems were in place in any of the study facilities, and all notes were recorded on paper. Only half of the facilities in our study conducted regular perinatal morbidity and mortality audits. Investigations or audits of perinatal morbidity and mortality cases are important and provide an opportunity for clinicians to understand more about individual cases, gain insights into avoidable practice and systems issues, drive changes in clinical practice and stimulate quality improvement initiatives [21, 62–64]. In addition, this information can be used to instigate research and inform public health initiatives to avoid future preventable newborn deaths and stillbirths [62, 64, 65]. Another study in the region reported on the evaluation of a program to improve perinatal mortality review systems in three Fijian hospitals, and identified that inadequate staffing, problems with medical equipment and a lack of clinical skills were significant contributors to newborn death [66]. The study noted that enabling factors for perinatal audits included leadership, teamwork and standardised processes [66].

Available tools to measure the quality of maternal and newborn care often focus on input measures alone, such as, the availability of essential medicines, equipment and other resources [67]. Measuring input measures alone does not give a complete picture of the care provided. For example, the availability of neonatal bag and masks does not indicate whether it was used correctly (or at all) for newborn resuscitation or made a difference to morbidity or mortality. Measures in real time of clinical actions are important for not only giving a more comprehensive assessment of the care provided but for examining the experience of care [67]. In our study, we conducted labour observations and interviews with women to understand newborn care experiences. Although resource-intensive, these measures are essential for informing quality improvement projects which address the provision and experience of care, as well as identifying efforts needed to progress towards more person-centred, respectful maternity services [67].

Multiple measures which examine both the provision and experience of care enable a greater understanding of what actually happens on the ground when babies are born in health facilities and can inform the development of effective interventions to improve the quality of newborn care. Through labour observations and interviews, we were able to examine early essential newborn care practices. We also observed whether efforts were made to keep mothers and babies together, a critical but underreported component of quality and respectful care of newborns [68]. The benefits of these early essential newborn practices are significant. Our finding that 60% of newborns initiated breastfeeding during this time is higher than global averages which have found that around 42% of newborns are put to the breast within the first hour of life [69]. Moreover, it is encouraging that 90% of the babies observed in our study received immediate skin-to-skin contact given the multiple benefits of skin-to-skin contact including temperature regulation and early initiation of breastfeeding [70–72]. Our findings are consistent with National Health Information System records for the same four facilities that report 92% of babies being breastfeed and 88% receiving skin-to-skin contact in the first hour. The high rates of these practices observed in this study is commendable and efforts to ensure that all newborns in PNG receive these four essential care practices to improve newborn health and survival should receive continued investment and be scaled-up [73]. In addition to these practices, it is equally important that training programs focus on the need to adequately prepare and assess the functionality of equipment prior to birth [74], which was observed to occur less than 50% of the time in our study.

### Implications for policy and practice

Measuring and describing the quality of newborn care provides an opportunity to clarify priority areas for newborn care improvement and provides the evidence for coordinated investment and action [67]. Global research continues to highlight that the biggest gains in newborn health outcomes will arise from concerted efforts to improve the quality of care during labour and immediately after birth [5]. In addition, efforts to improve newborn health needs to embrace the power of parents, families and communities in bringing about change [5]. The Every Newborn Action Plan [5] is a global commitment to preventing newborn deaths and stillbirths worldwide by 2030, building on the initial plan released in 2014 [19]. Critical components that need to be addressed to improve rates of newborn deaths and stillbirths are described and consistent with the factors identified in our study, such as quality of care, health workforce, medical commodities and technologies, and data for action [5].

Conducting facility audits and observing individual care practices in labour and immediately after birth, enables identification of specific areas where changes can be made to improve newborn care. Moreover, quality care teams have been established in four of the five participating facilities in East New Britain. These teams have actively drawn on the findings of this study to inform a series of facility-based quality improvement activities that, at the time of writing, are currently underway.

### Strengths and limitations

This study has several strengths. We used a co-designed mixed-methods approach whereby the study conception, design and implementation were co-developed with local stakeholders including the Provincial Health Authority, health providers and community members. Facility audits, labour observations and interviews with health providers and women enabled us to examine newborn care practices using multiple measures and triangulate findings. The small number of labour observations conducted (*N* = 30) provides a limited but important insight into newborn care practices in the first hour after birth. It is possible that the observer presence may have altered provider behavior. Given newborns were only observed for one hour after birth, mothers may have been provided with breastfeeding support and babies may have initiated breastfeeding outside of this one hour, which was not captured by our study. Newborns can initiate breastfeeding beyond one hour for multiple social, cultural and clinical factors [75], although the WHO recommends initiation of breastfeeding within the first hour for improved newborn health outcomes [9], including neonatal mortality [76]. Similarly, injections (Hepatitis B, Vitamin K and BCG) may have been given outside this period. It is important to note that many health providers in PNG have received training in early essential newborn care as part of initiatives rolled out by the WHO Western Regional Office [26, 30], however we did not collected data to determine which health providers observed, if any, had received this training. Whilst our study was conducted in one rural province in the Islands region of PNG, the findings are relevant to other regions and provinces of PNG, and there are important lessons for future newborn care research and program development in PNG and across the Pacific Islands region.

## Conclusion

Reductions in perinatal death rates in PNG requires renewed investment in interventions to improve the quality of newborn care, especially during labour, birth and the first day and week of life. Using a mixed-methods approach, we identified elements of early newborn care that are consistently implemented across different facility levels in East New Britain, PNG, including skin-to-skin, immediate drying, delayed cord clamping, and provision of Hepatitis B vaccination and Vitamin K. However, multiple barriers to improving the quality of newborn care were identified and include workforce shortages, infrastructure and utility constraints, and limited availability of newborn medicines and equipment. Measuring the quality of newborn care is the first step towards ensuring that all women and newborns are provided with high quality care, which in turn will lead to reduced newborn morbidity, mortality and stillbirths. Quality improvement interventions are needed at all levels of the health system - at the bedside, in the health facility and community through to the wider socio-political environment [5].

## Supplementary Information

**Additional file 1.**

**Additional file 2.**

**Additional file 3.**

**Additional file 4.**

## Data Availability

The datasets generated and/or analysed during the current study are not publicly available due to potential confidentiality concerns. Additional information can be made available from the Scientific Integrity Officer at Burnet Institute, (admin@burnet.edu.au), on reasonable request.
