# Supplementary material for: Quality newborn care in East New Britain, Papua New Guinea: measuring early newborn care practices and identifying opportunities for improvement
Source: BMC Pregnancy Childbirth. 2022 Jun 1;22:462. doi: 10.1186/s12884-022-04735-7 (PMC9157041; doi:10.1186/s12884-022-04735-7)
Supplement: Supplementary file 1 — Additional file 1. [file 12884_2022_4735_MOESM1_ESM.docx]

**Supplementary document 1. Facility Audit**

1. **Data collection details**

- Audit date: (Date)
- Research officer name
  - Facility name

1. Number of births/month: (number)
2. Number of births/month to young women (i.e. under 18 years)? (number)
3. Does the facility have capacity to give IV antibiotics?
   - Yes
   - No
4. Does the facility have capacity to give uterotonic drugs (i.e. IV oxytocin)?
   - Yes
   - No
5. Does the facility have capacity to give IV anticonvulsants for pre-eclampsia and eclampsia (i.e. magnesium sulfate)?
   - Yes
   - No
6. Does the facility have capacity to manually remove the placenta?
   - Yes
   - No
7. Does the facility have capacity to remove retained products (i.e. manual vacuum extraction, dilatation and curettage)?
   - Yes
   - No
8. Does the facility have capacity to perform assisted vaginal delivery (i.e. vacuum extraction, forceps delivery)?
   - Yes
   - No
9. Does the facility have capacity to perform basic neonatal resuscitation (i.e. with bag and mask)?
   - Yes
   - No
10. Does the facility have capacity to perform caesarean section?
    - Yes
    - No
    - If yes, number of caesareans/month (number)
11. Does the facility have capacity to perform blood transfusion?
    - Yes
    - No
12. Cost of vaginal birth to the patient:
    - (Kina)
13. Cost of caesarean birth to the patient:
    - (Kina)
    - Not applicable
14. Are there additional costs that a women is expected to pay related to in-patient stay? (i.e. bed linen, food, cleaning, nappies etc). Please describe [Free text]

**Facility units:**

1. Antenatal ward:
   - Yes
   - No
2. Labour ward:
   - Yes
   - No
3. Postnatal ward:
   - Yes
   - No
4. Combined antenatal and postnatal ward:
   - Yes
   - No
5. Special Care Nursery:
   - Yes
   - No
6. Newborn ward:
   - Yes
   - No
7. Dedicated outpatient clinic for antenatal patients?
   - Yes
   - No
8. Dedicated outpatient clinic for postnatal patients?
   - Yes
   - No
   - If not, where do re-admissions in the early postpartum period come to?

**Workforce**

1. How many doctors are currently working in maternity services? (number)
2. How many doctors are currently working in paediatrics? (number)
   - In newborn care? (number)
3. How many midwives? (number)
4. How many HEOs working in maternity services? (number)
5. How many general nursing officers working in maternity services? (number)
6. How many CHWs working in maternity services? (number)
7. Rostering and shifts (labour ward and birth suite)
   - Does the facility close after hours or is it open 24 hours?
   - Facility is open at set times (Please describe)
   - Facility is open 24 hours
8. How many shifts are there per day and what hours? (If these wards/clinics are not present, list as N/A) (free text)
   - Labour ward
   - Birth suite
   - Antenatal clinic
   - Antenatal ward
   - Postnatal ward
   - Neonatal ward
9. How many healthcare workers are rostered per shift in labour ward/birth suite?
   - Number of obstetricians per shift in labour ward/birth suite? (number)
   - Number of medical officers/junior doctors per shift in labour ward/birth suite? (number)
   - Number of midwives per shift in labour ward/birth suite? (number)
   - Number of HEOs per shift in labour ward/birth suite? (number)
   - Number of CHWs per shift in labour ward/birth suite? (number)

**Audit details:**

1. Does the Obstetrics and Gynaecology team met to discuss perinatal morbidity and mortality cases?
   - Yes
   - No
2. Are the meetings documented?
   - Yes
   - No
   - If yes, ask if copies are available
3. How often do they meet?
   - Weekly
   - Monthly
   - Every 3 months
   - Every 6 months
   - Once a year
4. Does the Paediatrics team met to discuss perinatal morbidity and mortality cases?
   - Yes
   - No
5. Are the meetings documented?
   - Yes
   - No
   - If yes, ask if copies are available
6. How often do they meet?
   - Weekly
   - Monthly
   - Every 3 months
   - Every 6 months
   - Once a year
7. Number of maternal deaths reviewed in past 12 months
   - How many were reviewed?
8. Number of newborn deaths reviewed in past 12 months
   - How many were reviewed?
9. Number of stillbirths deaths reviewed in past 12 months
   - How many were reviewed?

**Post-abortion care services:**

1. Does this facility provide post-abortion (spontaneous) care services?
   - Yes
   - No
   - Other (Free text)
2. Does this facility provide post-abortion (induced) care services?
   - Yes
   - No
   - Other (Free text)
3. Do women come to this facility seeking this service?
   - Yes
   - No
   - Other (Free text)
   - If yes, what is provided to these women? i.e. nothing, information, referral, contraceptive counselling and services (Free text)
4. **Laboratory**

- Select which of the following tests can be performed by the laboratory:
  - - Full Blood Count (Hb, Platelets, WCC, Neutrophils, Eosinophils, Reticulocytes etc)
    - Liver Function Tests (ALP, GGT, AST, ALT, Bilirubin etc)
    - Renal Function Tests (Sodium, Potassium, Chloride, Bicarbonate, Urea, Creatinine etc)
    - Haemoglobin
    - Mean Cell Volume (MCV)
    - Haemocrit
    - Malaria RDT
    - HIV
    - Syphillis
    - Microscopy (Urine)

1. **Physical locations and buildings available for antenatal inpatient care:**

- Number of Beds
- Privacy i.e. are there curtains
- Facilities available to patients:
  - Waiting areas
  - Companion areas
  - Toilets
  - Showers
  - Space to cook/prepare food
  - Space for charging phones
- Any comments on the above facilities? i.e. are they multi-purpose areas or especially for mothers and families? Are they clean? Patterns of use?

1. **Physical locations and buildings available for labour and birth:**

- Number of Beds (number)
- Are maternity resuscitation beds available?
  - Yes
  - No
  - If yes, how many? (number)
- Are neonatal resuscitation beds available?
  - Yes
  - No
  - If yes, how many? (number)
- Privacy i.e. are there curtains? (Describe, free text)
- Facilities available to patients: (Select which applies)
  - Waiting areas
  - Companion areas
  - Toilets
  - Showers
  - Space to cook/prepare food
  - Space for charging phones
- Any comments on the above facilities? i.e. are they multi-purpose areas or especially for mothers and families? Are they clean? Are they used by patients? (Free text)

1. **Physical locations and buildings available for immediate postnatal care:**

- Number of Beds (number)
- Privacy i.e. are there curtains? (Describe, free text)
- Facilities available to patients: (Select which applies)
  - Waiting areas
  - Companion areas
  - Toilets
  - Showers
  - Space to cook/prepare food
  - Space for charging phones
- Any comments on the above facilities? i.e. are they multi-purpose areas or especially for mothers and families? Are they clean? Patterns of use? (Free text)
- Are infant formula company materials visible? (posters, brochures, stickers, painted walls) (Describe, free text)
- Do mothers receive any products or gifts from baby-food companies? (Describe, free text)

1. Blood transfusion facilities?
   - Yes
   - No
   - Sometimes (please explain)
   - Other (please explain)
2. Hygiene and sanitation (confirm availability through visual inspection) (delivery room/newborn unit): (Select all that apply)
   - Running water
   - Soap
   - Disposable paper towels
   - Individual reusable hand towels
   - Alcohol hand rub/sanitizer
   - Waste disposal (waste pit, placenta pit, incinerator, burning) (Please describe)
   - Other (Free text)
3. Power supply
   - Lighting source (Select all that are available and fully functional)
     - National grid
     - Generator
     - Solar power
     - Other (Free text)
4. Availability of appropriate personal protective equipment (PPE) for health workers (i.e. masks, gowns, gloves) (Free text)
5. Availability of job aids i.e. laminated charts, wall charts, posters, leaflets etc appropriately place in a visible location: (Select all that apply)
   - Hand washing
   - Management of post-partum haemorrhage (PPH)
   - Active management of third stage of labour
   - Management of pre-eclampsia/eclampsia
   - Newborn resuscitation
   - Protocol for assisted vaginal delivery
   - Management of puerperal sepsis
   - Kangaroo Mother Care
   - Management of neonatal sepsis
   - Breastfeeding
6. Availability of the following maternal, adolescent and newborn guidelines i.e. are hard copies of these available in the facility? (Select all that apply)):
   - Manual of family planning
   - WHO family planning
   - Emergency Obstetric Care
   - Manual of Standard Management in O&G
   - Paediatrics for Doctors in PNG
   - Standard Treatment for Common Illnesses of Children
   - Standard Management of STIs
   - Minimum Standard Management of STI Services
   - Other [please specify]
7. Does the health facility have a written policy around infant formula use?
   - Yes
   - No
   - Other (Free text)
8. At what age does a woman need parental or guardian consent to receive treatment? (Number)
9. **Medicines and Supplies**

| **Medicines** | **Availability** | | | **Drugs within expiry date?** | |
| --- | --- | --- | --- | --- | --- |
|  | **Yes** | **No** | **Sometimes** | **Yes** | **No** |
| Amoxycillin |  |  |  |  |  |
| Antibiotic eye ointment application |  |  |  |  |  |
| Artemether-lumefantrine (Mala-1/Coartem) |  |  |  |  |  |
| Augmentin DF |  |  |  |  |  |
| Azithromycin |  |  |  |  |  |
| BCG vaccine |  |  |  |  |  |
| Benzyl-penicllin |  |  |  |  |  |
| Betamethasone |  |  |  |  |  |
| Ceftriaxone IV |  |  |  |  |  |
| Cephalexin |  |  |  |  |  |
| Chloroquine |  |  |  |  |  |
| Ciprofloxacin |  |  |  |  |  |
| Clotrimazole |  |  |  |  |  |
| dexamethasone |  |  |  |  |  |
| Doxycyline |  |  |  |  |  |
| Erythromycin |  |  |  |  |  |
| First-line anti-retroviral treatment i.e.  Tenofovir disoproxil fumarate (TDF), Lamivudine (3TC) or Emtricitabine (FTC), efavirenz (EFV) (Tick yes if any of these three are available) |  |  |  |  |  |
| Flucloxacillin |  |  |  |  |  |
| Gentamicin IV |  |  |  |  |  |
| Hepatitis B vaccine |  |  |  |  |  |
| HIV test kits |  |  |  | N/A | N/A |
| Influenza vaccine |  |  |  |  |  |
| Injectable antibiotics for management of newborn sepsis |  |  |  |  |  |
| Iron/folate supplement |  |  |  |  |  |
| Lignocaine |  |  |  |  |  |
| Magnesium sulphate |  |  |  |  |  |
| Methyl dopa or labetalol or nifedipine or hydralazine |  |  |  |  |  |
| Metronidazole IV |  |  |  |  |  |
| Misoprostol |  |  |  |  |  |
| Oxytocin IM or IV |  |  |  |  |  |
| Penicillin IV |  |  |  |  |  |
| Sulfadoxine-pyrimethamine |  |  |  |  |  |
| Syphilis test kits |  |  |  | N/A | N/A |
| Tetnaus vaccine |  |  |  |  |  |
| Umbilical chlorhexidine |  |  |  |  |  |
| Vitamin K |  |  |  |  |  |

1. **Equipment (Tick if yes)**

|  |  | | | **Available on?** | |  |  |
| --- | --- | --- | --- | --- | --- | --- | --- |
| **Equipment** | **Functional** | | | **Maternity/Gynae Unit** | **Newborn unit** | **Store** | **Other** |
|  | **Never** | **Sometimes** | **Always** |  |  |  |  |
| Adult weighing scales  *Check scale against calibrated weight |  |  |  |  |  |  |  |
| Airways (different sizes) |  |  |  |  |  |  |  |
| Artery forceps straight |  |  |  |  |  |  |  |
| Baby weighing scales  *Check scale against calibrated weight |  |  |  |  |  |  |  |
| Bag valve mask (adult) |  |  |  |  |  |  |  |
| Bowls |  |  |  |  |  |  |  |
| Caesarean section set |  |  |  |  |  |  |  |
| Cold box (for vaccines/medicines) |  |  |  |  |  |  |  |
| Colour coded bins  *for different waste products |  |  |  |  |  |  |  |
| Continuous positive airway pressure (CPAP) for newborns |  |  |  |  |  |  |  |
| Cord scissors |  |  |  |  |  |  |  |
| Doppler |  |  |  |  |  |  |  |
| Episiotomy scissors |  |  |  |  |  |  |  |
| Forceps |  |  |  |  |  |  |  |
| Functional autoclave or central sterilization services |  |  |  |  |  |  |  |
| Gynaecological examination light |  |  |  |  |  |  |  |
| Instrument tray |  |  |  |  |  |  |  |
| Kidney dish |  |  |  |  |  |  |  |
| Manual Vacuum Aspiration kit (for post abortion care) |  |  |  |  |  |  |  |
| Needle holder |  |  |  |  |  |  |  |
| Newborn ambu bag (self inflating) |  |  |  |  |  |  |  |
| Newborn resuscitaire |  |  |  |  |  |  |  |
| Flat surface to resuscitate? |  |  |  |  |  |  |  |
| Light? |  |  |  |  |  |  |  |
| Timer? |  |  |  |  |  |  |  |
| Warmer? |  |  |  |  |  |  |  |
| Newborn towels |  |  |  |  |  |  |  |
| Oxygen for newborn use |  |  |  |  |  |  |  |
| Pinards |  |  |  |  |  |  |  |
| Preterm and term masks (size 0 and 1) |  |  |  |  |  |  |  |
| Refrigerator (for vaccines/medicines) |  |  |  |  |  |  |  |
| Sphygmomanometer |  |  |  |  |  |  |  |
| Sterile gloves |  |  |  |  |  |  |  |
| Stethoscope |  |  |  |  |  |  |  |
| Suction device |  |  |  |  |  |  |  |
| Suction machine (manual/electrical) |  |  |  |  |  |  |  |
| Tendon hammer |  |  |  |  |  |  |  |
| Thermometer |  |  |  |  |  |  |  |
| Urine dipstick |  |  |  |  |  |  |  |
| Vacuum extractor |  |  |  |  |  |  |  |
| Vaginal speculums (any bivalve) |  |  |  |  |  |  |  |
