# Supplementary material for: Quality newborn care in East New Britain, Papua New Guinea: measuring early newborn care practices and identifying opportunities for improvement
Source: BMC Pregnancy Childbirth. 2022 Jun 1;22:462. doi: 10.1186/s12884-022-04735-7 (PMC9157041; doi:10.1186/s12884-022-04735-7)
Supplement: Supplementary file 2 — Additional file 2. [file 12884_2022_4735_MOESM2_ESM.pdf]

Labour Observation

Record ID

____

Instructions:
Greet the facility Sister in charge (SIC)/Officer in charge (OIC). Brief them about the observation and gain their consent. Provide them with information about the study.

Key points to include:

• This study is supported by the East New Britain Provincial Government and National Department of Health
• All efforts are made to ensure answers are anonymous
• Information collected will be used to improve planning and support of health services
• Burnet Institute and PNG IMR are conducting the study.

Ask the staff in labour ward/birth suite about any women in labour and to notify you of any pending births. The aim of the labour observation is to observe pregnant women in established labour who are giving birth in study facilities. Women will be observed throughout their labour, childbirth and until 1 hour postpartum (within working hours).

Move about the room to clear a clear view of the woman without obstructing the birth attendant(s), speaking or intervening.*

If questioned by patient family - please provide Tok Orait Pepa for information sheet use.

Pre-screening eligibility questions:

1. Date:

____

2. Woman initials:

____

3. Hospital:

◯ Nonga ◯ St Mary's ◯ Kerevat ◯ Napapar ◯ Pomio ◯ Other

Others, please specify.

____

4. Woman's age (as recorded in medical record):
Women under 16 years are ineligible

____

5. Woman admitted for childbirth

◯ Yes ◯ No (If not, not eligible)

6. Woman transferred to another hospital immediately, or taken straight to theatre (for caesarean section)?

◯ Yes (If yes, not eligible) ◯ No

7. Woman is presenting for abortion or abortion-related complications, miscarriage, fetal death in-utero, ectopic pregnancy, antenatal or postnatal complications:

◯ Yes (If yes, not eligible) ◯ No

8. Pre-screening eligibility. Is the women eligible?

◯ Yes ◯ No

9. Consent received from sister in charge/officer in charge.

◯ Yes ◯ No

General information about patient

10. Date observation commenced:

____

11. Time observation commenced:

____

12. Date and time of admission to labour ward:

____

Obstetric and medical characteristics (Collect from patient record):

13. Number of pregnancies including current:

____

14. Number of previous births excluding current:

____

15. Number of previous caesarean sections (if any):

____

16. Current pregnancy:

◯ Singleton ◯ Multiple

17. HIV status

◯ Negative ◯ Positive ◯ Unknown

Pre-Birth Preparation

18. Did the accoucher take actions to consider the room temperature? (i.e. turn off fans/air conditioning):

◯ Yes ◯ No ◯ Don't know

19. Did the accoucher wash their hands prior to touching any sterile/clean surface areas/handling equipment?

◯ Yes ◯ No ◯ Don't know

20. Did the accoucher prepare the newborn resuscitation area?

◯ Yes ◯ No ◯ Don't know

21. Did the accoucher check if newborn bag and basks were functional?

◯ Yes ◯ No ◯ Don't know

22. Did the accoucher arrange equipment for the birth - i.e. forceps, cord clamp/ties etc?

◯ Yes ◯ No

Birth

23. Was the labour spontaneous or induced?

◯ Spontaneous ◯ Induced

24. Was augmentation of labour used (artificial rupture of membranes or an oxytocin drip to speed up labour)?

◯ No ◯ Yes ◯ Unknown

25. Was perineal shaving performed?

◯ No ◯ Yes ◯ Unknown

26. Was an enema performed?

◯ No ◯ Yes ◯ Unknown

27. Did the woman have an episiotomy?

◯ Yes ◯ No (Go to 29)

28. If yes, was the procedure explained and woman agreed to the procedure?

◯ Not explained, did not agree ◯ Not explained, agreed ◯ Explained, did not agree
◯ Explained and agreed ◯ Refused procedure ◯ Unknown

29. Did a perineal tear occur?

◯ No tear (Skip to 32) ◯ First degree tear ◯ Second degree tear ◯ Third- or fourth-degree tear
◯ Yes but not sure which type ◯ Unknown

30. Perineal repair or suture performed?

◯ No ◯ Yes ◯ Unknown

31. Local anaesthetic used during perineal repair

◯ No ◯ Yes ◯ Unknown

32. Was the woman told she could move around and get off the bed during labour?

◯ Not told, did not mobilize ◯ Not told, mobilized ◯ Told, did not mobilize ◯ Told and mobilized
◯ Unknown

33. Was the woman instructed to clean up her own blood, urine, faeces or amniotic fluid?

◯ No ◯ Yes ◯ Unknown

34. Was the woman's companion or family members instructed to clean up her blood, urine, faeces or amniotic fluid?

◯ No ◯ Yes ◯ Unknown

35. Maternal admission to intensive care.

◯ No ◯ Yes ◯ Unknown

36. Maternal transfer to another hospital.

◯ No ◯ Yes ◯ Unknown

37. Maternal status at end of observation.

◯ Alive ◯ Dead ◯ Unknown

38. Mode of childbirth

◯ Non-instrumental vaginal birth (Skip to 40)
◯ Instrumental vaginal birth (vacuum/forceps) (Skip to 41)
◯ Caesarean section (Skip to 39)

39. If yes, was the procedure (caesarean section) explained and woman agreed to the procedure?

◯ Not explained, did not agree
◯ Not explained, agreed
◯ Explained, did not agree
◯ Explained and agreed
◯ Refused procedure
◯ Unknown

40. For women with a non-instrumental vaginal birth (ie. normal birth), what was the birth position?

◯ Dorsal/supine (flat on her back)
◯ Lithotomy (flat on her back with her feet in the stirrups)
◯ On all fours (hands and knees position)
◯ Squatting or sitting
◯ Lying on her side
◯ Unknown
◯ Other (please specify)

Others, please specify.

____

41. Total # of vaginal exams observed: (number)

____

42. Before a vaginal examination, did the staff inform the woman why a vaginal exam was needed and obtained her permission?

◯ Did not have any vaginal exam during observation period
◯ Not informed, permission not obtained
◯ Not informed, permission obtained
◯ Informed, permission not obtained
◯ Informed and permission obtained
◯ Refused examination
◯ Unknown

Immediate Postpartum Newborn activities

43. Singleton or multiple birth

◯ Singleton (1 baby)
◯ Multiples (2 or more babies)
◯ Unknown/don't know/missing

44 a) Sex of the baby (baby 1)

◯ Female
◯ Male
◯ Intersex
◯ Unknown

45 a). Baby status at birth (baby 1)

◯ Baby alive at birth (Skip to 48)
◯ Fresh stillbirth (skip to 46 & 47)
◯ Macerated stillbirth (baby has peeling skin) (Skip to 46 & 47)
◯ Unknown/don't know

46 a) Was the mother informed that her baby was stillborn or had died? (Baby 1)

◯ Yes ◯ No ◯ Unknown

47 a) Was the mother able to see the deceased or stillborn baby if she wished to? (Baby 1)

◯ Yes ◯ No ◯ Unknown

48 a) Did the baby start breathing on their own ? (Baby 1)

◯ Yes ◯ No, needed assistance

49 a) What assistance was needed? (Baby 1)

◯ Stimulation (rubbing/drying with cloth) ◯ Suctioning of mouth/nose ◯ Baby needed artificial breaths (mouth to mask or bag and mask) ◯ Other measures than those listed above (Please specify)

Others, please specify.

____

50 a) Was the baby dried after birth? (Baby 1)

◯ Yes ◯ No ◯ Unknown

51 a) Was the baby placed on mother's chest (direct skin-to-skin)? (Baby 1)

◯ Yes ◯ No ◯ Unknown

52 a) Was the baby covered with a cloth or blanket? (Baby 1)

◯ Yes ◯ No ◯ Unknown

53 a) Was the mother injected with oxytocin after the birth of the baby? (Baby 1)

◯ Yes ◯ No ◯ Unknown

54 a) Did the accoucher check for cord pulsations before clamping the umbilical cord, clamped after pulsations stopped? (Baby 1)

◯ Yes ◯ No ◯ Unknown

55 a) Was the mother encouraged to start breastfeeding? (Baby 1)

◯ Yes ◯ No ◯ Unsure

56 a) Did the baby start breastfeeding? (Baby 1)

◯ Yes ◯ No ◯ Unsure

57 a) Was breastfeeding advice provided to the mother? (baby 1)

◯ Yes ◯ No ◯ Unsure

58 a) Was the baby given Vit K? (Baby 1)

◯ Yes ◯ No ◯ Unsure

59 a) Was the baby given Hep B? (baby 1)

◯ Yes ◯ No ◯ Unsure

60 a) Baby status at end of observation period. (Baby 1)

◯ Alive and well ◯ Baby admitted to special care baby unit ◯ Very early infant death (birth to 1 hour postpartum)

61 a) During the time of observation after birth where was the baby for most of the time? (Baby 1)

☐ Held by the mother, skin to skin
☐ Held by the mother, not skin to skin
☐ On the mother's bed
☐ Held by a family member
☐ Held by a birth attendant
☐ In a cot in the same room as the mother, within mother's reach.
☐ In a cot in the same room as the mother, not in mother's reach.
☐ On a neonatal resuscitaire
☐ In a neonatal nursery
☐ Other (please specify)

Others, please specify.

____

44 b) Sex of the baby (baby 2)

◯ Female
◯ Male
◯ Intersex
◯ Unknown

45 b). Baby status at birth (baby 2)

◯ Baby alive at birth (Skip to 48)
◯ Fresh stillbirth (skip to 46 & 47)
◯ Macerated stillbirth (baby has peeling skin) (skip to 46 & 47)
◯ Unknown/don't know

46 b) Was the mother informed that her baby was stillborn or had died? (Baby 2)

◯ Yes ◯ No ◯ Unknown

47 b) Was the mother able to see the deceased or stillborn baby if she wished to? (Baby 2)

◯ Yes ◯ No ◯ Unknown

48 b) Did the baby start breathing on their own ? (Baby 2)

◯ Yes ◯ No, needed assistance

49 b) What assistance was needed? (Baby 2)

○ Stimulation (rubbing/drying with cloth) ○ Suctioning of mouth/nose ○ Baby needed artificial breaths (mouth to mask or bag and mask) ○ Other measures than those listed above (Please specify)

Others, please specify.

____

50 b) Was the baby dried after birth? (Baby 2)

○ Yes ○ No ○ Unknown

51 b) Was the baby placed on mother's chest (direct skin-to-skin)? (Baby 2)

○ Yes ○ No ○ Unknown

52 b) Was the baby covered with a cloth or blanket? (Baby 2)

○ Yes ○ No ○ Unknown

53 b) Was the mother injected with oxytocin after the birth of the baby? (Baby 2)

○ Yes ○ No ○ Unknown

54 b) Did the accoucher check for cord pulsations before clamping the umbilical cord, clamped after pulsations stopped? (Baby 2)

○ Yes ○ No ○ Unknown

55 b) Was the mother encouraged to start breastfeeding? (Baby 2)

○ Yes ○ No ○ Unsure

56 b) Did the baby start breastfeeding? (Baby 2)

○ Yes ○ No ○ Unsure

57 b) Was breastfeeding advice provided to the mother? (baby 2)

○ Yes ○ No ○ Unsure

58 b) Was the baby given Vit K? (Baby 2)

○ Yes ○ No ○ Unsure

59 b) Was the baby given Hep B? (baby 2)

○ Yes ○ No ○ Unsure

60 b) Baby status at end of observation period. (Baby 2)

○ Alive and well ○ Baby admitted to special care baby unit ○ Very early infant death (birth to 1 hour postpartum)

61 b) During the time of observation after birth where was the baby for most of the time? (Baby 2)

☐ Held by the mother, skin to skin
☐ Held by the mother, not skin to skin
☐ On the mother's bed
☐ Held by a family member
☐ Held by a birth attendant
☐ In a cot in the same room as the mother, within mother's reach.
☐ In a cot in the same room as the mother, not in mother's reach.
☐ On a neonatal resuscitaire
☐ In a neonatal nursery
☐ Other (please specify)

Others, please specify.

____

The next section of the observation may be performed outside of the labour room/birth suite. Take the time to sit down and think through the events observed and respond based on what you saw and heard

Respectful and dignified care

62. Did the woman have a birth attendant present during labour?

◯ Yes - all of the time ◯ Yes - some of the time ◯ No

63. If yes, who was the attendant? (Tick all that apply)

☐ Midwife
☐ Doctor
☐ Nurse
☐ HEO
☐ CHW
☐ Midwifery student
☐ Nursing student
☐ Medical student
☐ Don't know who they were

64. Did the woman have a birth attendant present when the baby was actually born?

◯ Yes ◯ No

65. If yes, who was the attendant?

◯ Midwife
◯ Doctor
◯ Nurse
◯ HEO
◯ CHW
◯ Not sure

66. Did the birth attendant introduce themselves to the woman ?

◯ Yes ◯ No ◯ Unsure

67. Did the birth attendant explain what was happening/provide information to the women at any stage during the labour or birth?

◯ Yes ◯ No ◯ Unsure

68. Did the birth attendant invite the woman to ask any questions any stage during the labour or birth?

◯ Yes ◯ No ◯ Unsure

69. Did the birth attendant encourage the woman during the labour?

◯ Yes ◯ No ◯ Unsure

70. Was the woman offered to have a labour companion during labour and birth:

◯ Yes ◯ No ◯ Unsure

71. Was there a companion present at any time during labour?

◯ Yes ◯ No ◯ Unsure

72. Was there a companion present at the time of birth?

◯ Yes ◯ No ◯ Unsure

73. Was the women offered pain relief and did she receive it?

◯ Offered and received ◯ Offered and not received ◯ Not offered ◯ Unknown

74. Did the woman request pain relief?

◯ Requested, not received ◯ Requested and received ◯ Not requested ◯ Unknown

75. Was the woman asked for her preferred birthing position?

◯ Yes ◯ No ◯ Unsure

76. Were curtains, partitions, or other measures used to provide privacy for the woman throughout labour, childbirth and postpartum periods?

◯ Yes - it was private ◯ Yes - but the curtains did not close and could not provide privacy
◯ No ◯ Used during some but not all periods

77. Was the women offered any water or oral fluids during labour?

◯ No ◯ Yes ◯ Unknown

78. Did the woman have easy access to water or oral fluids during labour?

◯ No ◯ Yes ◯ Unknown

79. Was there easy access to toilet and shower facilities (i.e. in room, next to room)?

◯ Yes ◯ No

80. Any physical abuse? (tick all that apply)

- ☐ Slap
- ☐ Forceful downward pressure on abdomen
- ☐ Held down to the bed forcefully
- ☐ Punch
- ☐ Hit
- ☐ Kick
- ☐ Pinch
- ☐ Gag
- ☐ Tied to the bed
- ☐ Other physical abuse

81. Any verbal abuse? (tick all that apply)

- ☐ Shouted at
- ☐ Scolded
- ☐ Mocked
- ☐ Insulted
- ☐ Threatened with poor outcome for baby
- ☐ Hissed at
- ☐ Negative comments - her sexual activity
- ☐ Threatened with medical procedure
- ☐ Negative comments - her appearance
- ☐ Threatened to withhold care
- ☐ Negative comments - baby's appearance
- ☐ Threatened with physical violence
- ☐ Blamed woman for poor outcome

82. Any discrimination? (tick all that apply)

- ☐ Province of origin
- ☐ Economic circumstances
- ☐ In relation to her age, eg. being made to feel that she was too young or too old to have a baby
- ☐ Single status - eg. discriminated because she was not married
- ☐ Level of education/literacy
- ☐ Religion
- ☐ HIV status
- ☐ Other stigma/discrimination

Others, please specify.

____

Date observation ended:

____

Time observation ended:

____

Total time observed by research officer (mins):

____
